# Bridging Communication Gaps to Enhance Patient Safety: A Quality Improvement (QI) Project on the Role of Abbreviations, Their Risks, and Pathways to Change

**DOI:** 10.7759/cureus.95843

**Published:** 2025-10-31

**Authors:** Saarah Talha, Ben Smith, Ayesha Khan, Zaina Gaddoura

**Affiliations:** 1 Ear, Nose, and Throat (ENT), Royal Shrewsbury Hospital, Shrewsbury, GBR; 2 Orthopaedics, Royal Shrewsbury Hospital, Shrewsbury, GBR; 3 General Surgery, Royal Shrewsbury Hospital, Shrewsbury, GBR; 4 Orthopaedics, Royal Stoke University Hospital, Stoke-on-Trent, GBR

**Keywords:** clinical documentation improvement, documentation, ent surgeon, head neck surgery, professional communication, safety guidelines, safety patient, surgical notes

## Abstract

Introduction

Effective communication within the multidisciplinary team (MDT) is critical to safe patient care. Whilst electronic health records have improved legibility, the widespread use of non-standardised abbreviations continues to cause misinterpretation, risking delays, errors, and compromised patient safety. Abbreviations are often used for efficiency, yet their meanings vary between specialties, creating barriers for rotating staff, cross-disciplinary colleagues, and patients reviewing discharge summaries.

Quality improvement project

We conducted a closed-loop quality improvement (QI) project structured around the SQUIRE 2.0 guidelines for QI initiatives. The project took place in a UK district general hospital to assess and improve understanding of commonly used ear, nose, and throat (ENT) specialty abbreviations. Eighty-two common abbreviations were identified and used in a written expansion test. In cycle one (n = 45), mean accuracy was 24.3%, with no participant exceeding 77%. Following targeted interventions (educational seminars and a printed reference guide displayed in shared spaces), a second assessment cycle was conducted with a new cohort. Post-teaching scores improved by a relative 40% to a mean of 35% (range 24%-52%). Mann-Whitney U testing confirmed statistical significance (p < 0.05).

Implications and discussion

Findings revealed a substantial baseline knowledge gap, highlighting a safety risk. Low-cost, high-visibility measures, such as reference posters and induction-based teaching, improved comprehension and could be readily adopted in other departments. However, residual gaps suggest the need for upstream interventions, including integration of documentation clarity training into medical education and continued departmental reinforcement.

Conclusion

Clinicians’ baseline abbreviation comprehension was poor, but targeted education significantly improved understanding. Whilst no participant achieved complete proficiency, results show that simple, resource-light strategies can enhance communication clarity and support safer care. Broader adoption, alongside curricular change, offers a sustainable path to reducing abbreviation-related risks.

## Introduction

Effective communication is imperative to the function of the multidisciplinary team (MDT). Amongst the various modes of information exchange, documentation is one of the most important and can often serve as the primary form of communication within the MDT. The introduction of electronic health records has significantly improved legibility and accessibility of clinical notes. However, the content may still utilise specialist terminology that is not universally understood by all healthcare professionals [1]. A limiting factor to the success of electronic records is the ongoing prevalence of abbreviations. Whilst shorthand may enhance efficiency, its misinterpretation can contribute to miscommunication and potential harm [2].

The challenge of documentation ambiguity extends beyond intra-professional exchanges to the interface between clinicians and patients. Discharge summaries, which are intended to empower patients with knowledge of their treatment and post‐hospital care, frequently contain shorthand that acts as a barrier to comprehension [3]. Such obscurity may undermine adherence to follow-up instructions and erode patient trust in their healthcare providers [4,5].

Currently, healthcare professionals face heavy documentation demands and intense time pressures. This undoubtedly fuels abbreviation usage to accelerate documentation and conform to local conventions. Condensing terms such as ‘physiotherapy’ to ‘PT’ may offer marginal time savings, but without universally accepted standards, many abbreviations remain unclear. This can be particularly challenging for resident or locum doctors rotating into a new specialty. This lack of clarity has been linked to diagnostic delays or medication errors, all of which compromise patient safety [6]. Knowing the consequences of poor communication and the barrier posed by abbreviation usage, it is imperative to explore the drivers behind this practice and to develop strategies for ensuring clarity in medical documentation [7].

A clear understanding of exchanging information is essential to patient-care transition throughout admission and beyond. Abbreviations allow for efficiency; however, the brevity that expedites documentation can fracture communication when interpretations diverge [8]. The transient nature of clinical teams and the continual influx of rotating trainees further compound the challenge of maintaining clarity.

## Materials and methods

Quality improvement project 

In designing this project, we recognised that the convenience of using clinical abbreviations potentially conceals gaps in communication and compromises patient safety. To explore this, we designed a knowledge assessment to assess the degree of unfamiliarity with medical abbreviations amongst healthcare professionals (Appendices).

Methods

We conducted a two-cycle closed-loop quality improvement project designed using the SQUIRE 2.0 framework in a UK district general hospital [9]. The study was conducted over two four-month cycles, beginning in January 2024 and concluding in March 2025. An ear, nose, and throat (ENT) consultant body identified 82 abbreviations commonly encountered in ward rounds, operation notes, prescribing records, and multidisciplinary communications. A short-answer written assessment was created from this to identify the full form of each abbreviation. Participants were briefed on the purpose of the project before completing the assessment. Participation was voluntary, and all responses were anonymised.

During the 2024 cycle, 45 (n = 45) healthcare professionals completed the assessment. We assessed professionals involved in communicating or providing care for the patient, namely, nurses, resident doctors, administrative secretaries, ward clerks, physiotherapists, and advanced care practitioners. We excluded ENT registrars and consultants, whose expertise in departmental shorthand is already well established, whilst intentionally including senior clinicians from other specialties in order to capture the full breadth of multidisciplinary interactions. Responses were collated and scored out of 82. Answer spaces left blank were considered incorrect.

Following our first audit, we instituted educational interventions aimed at improving abbreviation recognition. We introduced a standardised reference document to combat communication barriers. The document was placed in common spaces throughout the department. Its purpose was twofold: to discourage the proliferation of new abbreviations and to provide a clear reference for those already prevalent in documentation.

The second cycle was completed the following year. Following an initial assessment at the rotating staff induction, a teaching seminar was delivered, discouraging abbreviation usage whilst highlighting the commonly used abbreviations. They were then re-assessed to measure their improvement in abbreviation recognition. The scores were calculated using the same criteria as in the first audit cycle.

## Results

The first audit cycle revealed a striking deficiency in abbreviation comprehension: participants correctly expanded only 24.3% of abbreviations (mean score 19.9/82), and no individual exceeded 77% accuracy. This uniformly low performance (even amongst those regularly exposed to ENT shorthand) highlighted a critical communication gap.

Following the intervention, pre-teaching performance in the second audit cycle showed a modest uplift from the initial mean score, with individual scores spanning 19.5% to 34.1%. Following the induction teaching, scores rose further, reflecting up to a 40% relative increase in mean scores from the first cycle to a mean of 35%, with post-teaching scores ranging from 23.2% to 52.4%.

**Figure 1:**
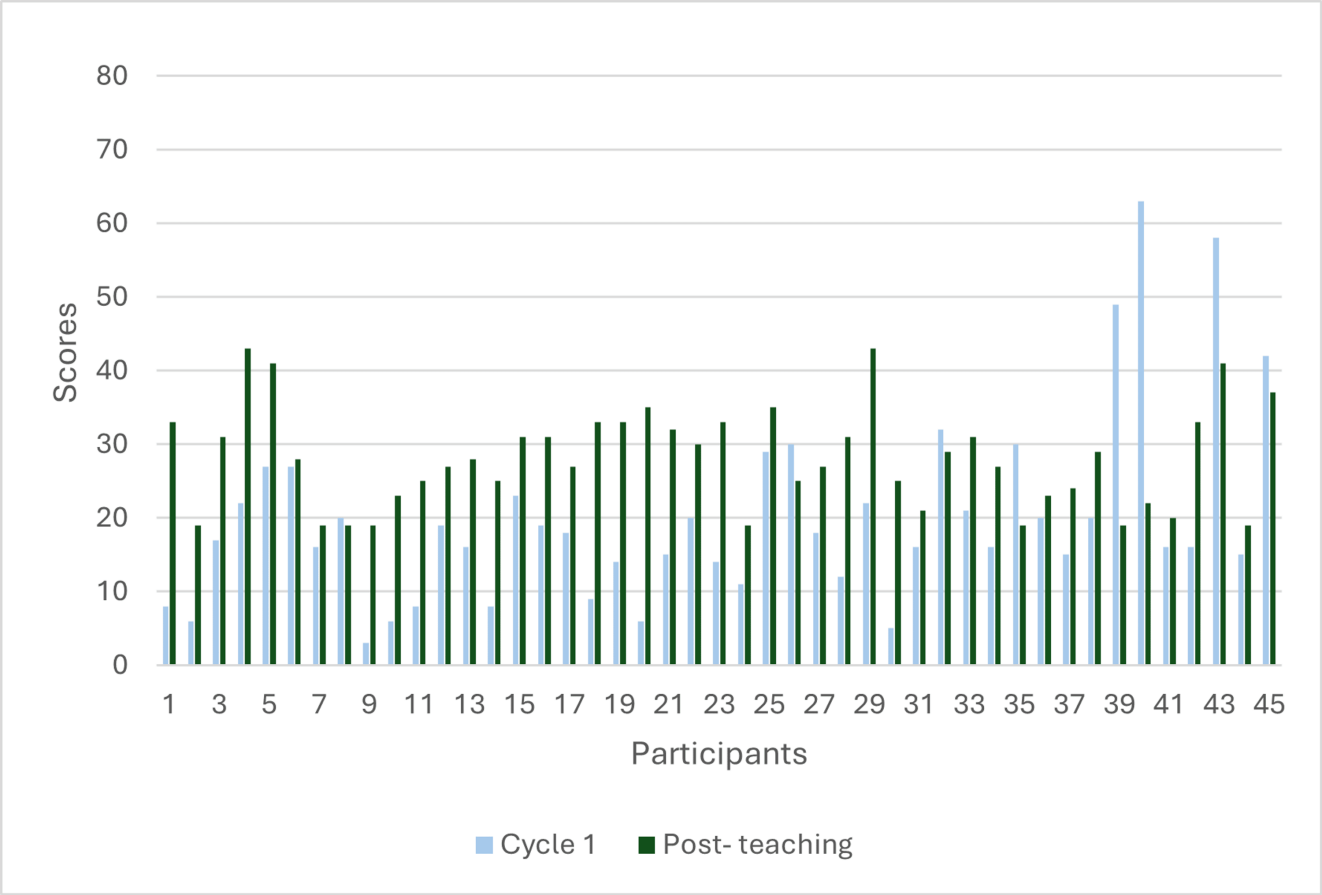
A bar chart demonstrating participants’ assessment scores between cycle 1 (pre-teaching) and post-teaching Scores of the 45 participants in cycle 1 (blue) and post-teaching (green) groups. The graph demonstrates a general consistent improvement in performance for most participants in the post-teaching group. Each bar represents an individual participant’s scores, with maximum scores being 82 per assessment.

A Mann-Whitney U test was used to analyse the statistical significance of the data. The non-parametric nature, along with it being unpaired data, was the reason this statistical test was used, which demonstrated a p-value of <0.05, which indicates the educational interventions significantly improved abbreviation recognition compared to cycle one (Tables 1-3; Figure 1).

**Table 1:** Cycle one (baseline) and pre-teaching (poster intervention) group assessment scores Comparison of assessment scores between the baseline (cycle one) and pre-teaching (poster) groups (N = 45 participants per group). Mann-Whitney U analysis showed no statistically significant difference between groups (p = 0.052; significance defined as p < 0.05).

Group	N	Mean score out of 82 (raw)	Range	U value	p-value
Cycle one (baseline)	45	19.93 (24.31%)	3.66%-76.83%	770.5	0.0503
Pre-teaching (posters)	45	20.51 (25.01%)	19.51%-34.15%		

**Table 2:** Pre-teaching (poster intervention) and post-teaching group assessment scores Comparison of assessment scores between the pre-teaching (poster) and post-teaching groups (N = 45 participants per group). Analysis using the Mann-Whitney U test indicated a statistically significant increase in post-teaching scores (p < 0.005).

Group	N	Mean score out of 82 (raw)	Range	U value	p-value
Pre-teaching (posters)	45	20.51 (25.01%)	19.51%-34.15%	338.5	<0.005
Post (teaching)	45	28.09 (34.25%)	23.17%-52.44%		

**Table 3:** Cycle one (baseline) and post-teaching group assessment scores Comparison of assessment scores between the baseline (cycle one) and post-teaching groups (N = 45 participants per group). Mann-Whitney U analysis indicated a statistically significant improvement following teaching (p < 0.005).

Group	N	Mean score out of 82 (raw)	Range	U value	p-value
Cycle one (baseline)	45	19.93 (24.31%)	3.66%-76.83%	413	<0.005
Post (teaching)	45	28.09 (34.25%)	23.17%-52.44%		

## Discussion

UK postgraduate training pathways are inherently rotational, with resident doctors often changing specialties, hospitals, or trusts every four, six, or 12 months. Within this context, our findings demonstrate that baseline knowledge of ENT abbreviations was limited, even amongst permanent departmental staff. Encouragingly, the introduction of standardised reference sheets and educational seminars led to improvement. These interventions remain straightforward and cost‐effective methods of enhancing patient care and improving interprofessional communication.

Importantly, the challenges identified are not unique to ENT. Similar trends have been observed elsewhere: Sheppard et al. reported comparable deficits in a paediatric department in the West Midlands, whilst Sinha et al. highlighted that surgical acronyms in general are poorly understood, particularly by foundation doctors and allied healthcare professionals [10,11]. The comparatively lower levels of awareness observed in our study may partly reflect the absence of a universally agreed list of acronyms. Variability between departments or trusts inevitably fosters divergent abbreviation practices, thereby compounding the potential for misunderstanding.

Although our intervention was applied department‐wide, certain subgroups would most likely benefit from access to reference tools-particularly secretarial staff and resident doctors responsible for drafting discharge summaries. Reducing ambiguity at the interface between hospital records and patient‐directed documentation is critical. Visual aids, such as index posters, have been shown to improve the accuracy and consistency of clinical documentation, thereby enhancing the clarity and utility of discharge summaries for both patients and community healthcare providers [12].

Despite the well‐recognised risks of abbreviation use, guidance from national regulatory bodies remains limited. In the UK, Good Medical Practice states documentation must be ‘clear, accurate, contemporaneous, and legible’ but does not explicitly address abbreviations [13]. By contrast, the U.S. Joint Commission offers brief recommendations regarding shorthand to avoid, though these are not specialty‐specific [14]. Given the time pressures and administrative burden inherent in modern clinical practice, the complete elimination of abbreviations is unrealistic. Instead, standardisation-coupled with targeted education-represents the most pragmatic way forward. What is more, embedding training on standardised documentation practices into undergraduate medical curricula may reduce the likelihood that cryptic shorthand becomes entrenched in clinical culture. Early exposure to the principles of clear, accessible record‐keeping could mitigate the perpetuation of unsafe habits.

To sustain and expand upon the improvements observed in this project, we propose retaining the low‐resource, high‐visibility elements of the intervention. Continuing to display reference posters and incorporating a brief abbreviation module into departmental inductions represent pragmatic and scalable approaches to reinforcing best practice amidst high staff turnover. Whilst such measures cannot eradicate the problem entirely, they offer practical reminders that support a culture of precision in clinical documentation. These local initiatives can complement wider curricular and policy reforms, ultimately strengthening patient safety across healthcare systems.

Limitations

The sample size here was modest, with 45 participants in each group. Whilst sufficient for initial analysis, a larger cohort would have increased the statistical power and improved the generalisability of the findings across different healthcare settings.

Moreover, there is a risk of sample bias due to variation in participants’ professional roles and levels of experience. Each group included a mix of doctors, nurses, and other practitioners, some of whom may have had prior exposure to ENT or greater familiarity with medical abbreviations. These differences were not controlled for and may have influenced performance independently of the intervention. Future studies could stratify participants or use matched groups to reduce this variability.

A further limitation lies in the test content. Since there is no nationally standardised list of ENT abbreviations, the test was developed with input from local ENT consultants. Whilst this ensured relevance within our department, it may reduce the external validity of the test in other contexts with different terminology practices.

Lastly, the sequential nature of the study design presents a potential source of bias. The control group completed the test before the intervention group, introducing the possibility that external factors between testing periods-such as clinical exposure or informal learning-could have influenced the outcomes. A randomised or simultaneous testing approach would help mitigate this in future research. To build on these findings, further studies could involve larger, more diverse cohorts across multiple centres and explore the development of a standardised abbreviation list to improve consistency and comparability.

## Conclusions

This study highlights the risks associated with inconsistent abbreviation use in clinical documentation. Simple, low-cost interventions such as reference posters and teaching sessions were effective in improving comprehension within the department and can be maintained with minimal resources. However, the lack of national standardisation means variability is likely to persist between trusts and specialties. Complete avoidance of abbreviations in time-pressured clinical environments is unrealistic; instead, combining standardisation with education at undergraduate, postgraduate, and departmental levels offers a practical path forward. By embedding clarity into documentation practices, healthcare organisations can strengthen communication, particularly at transition points of care, and thereby enhance patient safety.

## Figures and Tables

**Figure 2:**
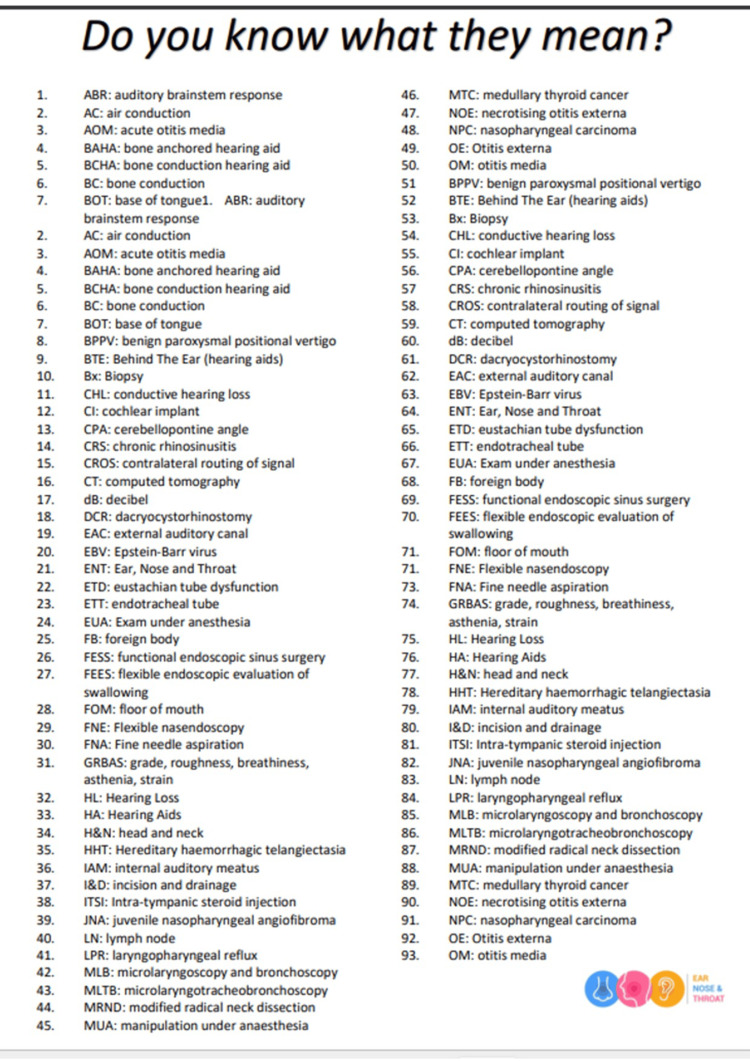
ENT department abbreviation reference document ENT: ear, nose, and throat
